# Comparison of recurrence patterns between patients with thoracic esophageal squamous cell carcinoma treated with neoadjuvant chemoradiotherapy and postoperative adjuvant chemoradiotherapy

**DOI:** 10.3389/fonc.2025.1604808

**Published:** 2025-06-16

**Authors:** Kunhan Ni, Yixuan Huang, Simiao Lu, Longlin Jiang, Huan Zhang, Wenwu He, Chenghao Wang, Qiang Zhou, Haojun Li, Jialong Li, Kangning Wang, Guangyuan Liu, Qiang Fang, Lin Peng, Xuefeng Leng, Yongtao Han

**Affiliations:** ^1^ Department of Thoracic Surgery, the Affiliated Hospital of Southwest Medical University, Sichuan, Luzhou, China; ^2^ Department of Thoracic Surgery, Sichuan Clinical Research Center for Cancer, Sichuan Cancer Hospital and Institute, Sichuan Cancer Center, University of Electronic Science and Technology of China (UESTC), Chengdu, China

**Keywords:** esophageal squamous cell carcinoma, esophagectomy, chemoradiotherapy, recurrence patterns, propensity score matching

## Abstract

**Purpose:**

To compare the recurrence patterns and survival outcomes between patients with esophageal squamous cell carcinoma (ESCC) treated with neoadjuvant chemoradiotherapy (NCRT) and adjuvant chemoradiotherapy (ACRT).

**Methods:**

We retrospectively analyzed 267 patients with locally advanced ESCC who received treatment at Sichuan Cancer Hospital and Institute (Chengdu, China) between January 2018 and December 2020. Based on different treatment protocols, the patients were divided into two groups: NCRT (n=181) and ACRT (n=86). After propensity score matching, each group included 74 patients. This study compared the recurrence types, sites, frequencies, and timing, as well as overall survival (OS), disease-free survival (DFS), and prognostic risk factors between the two groups.

**Results:**

The recurrence rates in the NCRT and ACRT groups were 59.5% (44/74) and 33.8% (25/74), respectively; the difference was statistically significant (P=0.002). Recurrences primarily occurred within 2 years following esophagectomy. The ACRT group had a higher 3-year OS rate than the NCRT group (67.8% versus [vs.] 50.6%, respectively; P=0.019). In the subgroup of patients with local recurrence, the 3-year OS rate was higher in the NCRT group compared to the ACRT group (53.8% vs. 0%, respectively; P=0.029). In terms of DFS, the ACRT group exhibited better results than the NCRT group (P<0.001). Multivariate analysis revealed that pathological N staging was an independent risk factor affecting the OS prognosis of patients in the NCRT group. Margin status and pathological T staging were identified as independent risk factors influencing OS in the ACRT group, while sex and treatment regimen were independent risk factors affecting DFS in patients with postoperative pathological lymph node positivity.

**Conclusion:**

There was significant difference in the OS and DFS prognosis of patients with ESCC treated with NCRT and ACRT. Recurrence primarily occurs within 2 years following esophagectomy. The recurrence rate was higher in the NCRT group compared to the ACRT group. Patients with early recurrence had a poorer survival prognosis compared to those with late recurrence. Pathological N staging was identified as an independent risk factor affecting OS in the NCRT group. Furthermore, margin status and pathological T staging were independent risk factors influencing OS in the ACRT group.

## 1 Introduction

Esophageal cancer is one of the most common types of digestive tract tumors. According to GLOBOCAN 2022, esophageal cancer ranks 11th and 7^th^ worldwide in terms of incidence and mortality (1). In 2015, Chinese Cancer Epidemiology Statistics reported approximately 246,000 new cases of esophageal cancer and 188,000 related deaths, accounting for nearly half of the global incidence and mortality rates. Squamous cell carcinoma accounted for >90% of these cases, predominantly affecting elderly males (2, 3).

Despite recent advances in treatment modalities, surgery remains the primary approach for the treatment of locally advanced esophageal squamous cell carcinoma (ESCC). Nevertheless, surgical intervention is associated with a low 5-year survival rate (20–33%) for patients with locally advanced ESCC (4–6). Due to the low cure rate and high recurrence rates after surgery, numerous randomized controlled trials have evaluated the effects of chemotherapy, radiotherapy, and surgery. Results from several large multicenter randomized controlled trials indicate that neoadjuvant chemoradiotherapy (NCRT) provides better outcomes compared to surgery alone, improving survival. The CROSS study (7) compared the efficacy of chemoradiotherapy combined with surgery versus (vs.) surgery alone for the treatment of esophageal cancer. The chemoradiotherapy regimen included weekly low-dose carboplatin and paclitaxel, alongside synchronous radiotherapy at 41.4 Gy, followed by surgical resection. The NCRT group demonstrated a higher R0 resection rate compared to the surgery-only group, along with improved median survival and 5-year survival rates (8).

In the NEOCRTEC5010 study (9), 451 patients with ESCC were randomly divided into NCRT and surgery-only groups. The NCRT group received two cycles of vinorelbine combined with cisplatin, alongside synchronous radiotherapy at 40.0 Gy. The postoperative pathological complete response rate in the NCRT group reached 43.2%. Moreover, the NCRT group had significantly higher R0 resection rate compared to the surgery-only group (98% vs. 91%, respectively; P=0.002). In terms of survival prognosis, the overall survival (OS) rate was significantly better in the NCRT group compared to the surgery-only group. Thus, NCRT is currently recommended as the first-line treatment for locally advanced ESCC.

Furthermore, studies have indicated that adjuvant chemoradiotherapy (ACRT) post-surgery can also enhance survival outcomes compared to surgery alone. A meta-analysis involving 13 studies and 2,165 patients showed (10) that ACRT significantly improved OS in patients with esophageal cancer and markedly reduced local recurrence rates. In another prospective study (11), 158 patients with esophageal cancer were randomly assigned to an ACRT group (n=78) and a surgery-only group (n=80). The ACRT group received two cycles of paclitaxel combined with cisplatin, with a total radiotherapy dose of 40 Gy. The results indicated that the median survival time, 10-year OS, and progression-free survival were significantly better in the ACRT group than the surgery-only group.

ESCC is characterized by high rates of local and distant disease recurrence. Regarding neoadjuvant therapy, preoperative chemoradiotherapy can downstage tumors, increase pathological complete response and R0 resection rates, and improve local and systemic control. However, the risk of recurrence remains high following esophagectomy, with rates exceeding 40% (12–15). Studies have investigated the patterns of recurrence following neoadjuvant treatment. The phase III clinical trial NEOCRTEC5010 (16) investigated the types, sites, frequency, timing, and prognostic factors of postoperative recurrence in esophageal cancer. After a median follow-up of 51.9 months, the recurrence rate was lower in the NCRT group than the surgery-only group (33.7% vs. 45.8%, respectively; P=0.013), with significant reductions noted in the rates of local recurrence and distant metastasis. Recurrence appeared later in the neoadjuvant group compared to the surgery-only group, particularly recurrence after 3 years (16.1% vs. 5.8%, respectively; P=0.029). The most common sites of distant metastasis in the NCRT group were the lungs, liver, pleura, and brain. In the surgery-only group, the most common sites were the lungs, bones, and liver. Prognostic analysis of disease-free survival (DFS) revealed that, in the NCRT group, the number of resected lymph nodes and pathological N stage were independent prognostic factors for recurrence. In the surgery-only group, R1 resection and pathological N1 stage significantly increased the risk of recurrence. Further analysis of the NEOCRTEC5010 trial (17) revealed significant discrepancies in the 5-year OS rates among patients with different types of recurrence. Specifically, the recurrence group had poorer prognosis than the non-recurrence group (17.8% vs. 89.2%, respectively; P<0.001), early recurrence was associated with worse outcomes compared to late recurrence (4.6% vs. 51.2%, respectively; P<0.001), and patients experiencing early recurrence had significantly shorter survival times following recurrence than those with late recurrence (P=0.028). However, there was no significant difference in post-recurrence survival between patients with distant metastasis and those with local recurrence (17.0% vs. 20.0%, respectively; P=0.666). Multivariate analysis indicated that pathological N1 stage, lymphadenectomy of <20 nodes, and lack of response to NCRT were independent risk factors for early postoperative recurrence.

Another study analyzing factors associated with local recurrence following NCRT (18) showed no difference in median survival times between local recurrence and distant metastasis. In multivariable analysis, clinical T staging and lack of response to neoadjuvant therapy emerged as independent risk factors for the prognosis of local recurrence, consistent with the findings of the 5010 study (17).

Unlike the recurrence patterns associated with neoadjuvant therapy, research on postoperative adjuvant therapy is currently limited. Studies indicated (13) that postoperative ACRT can prolong the median time to first recurrence. Moreover, another retrospective study that included only 31 cases treated with postoperative ACRT (19) demonstrated that the combination of postoperative ACRT with esophageal cancer resection could increase survival duration and DFS time. Although these studies provided evidence that postoperative ACRT improves recurrence outcomes compared to surgery alone, they did not specifically address squamous cell carcinoma and had small sample sizes. A retrospective study (20) included 1,390 patients with R0 resection of ESCC, consisting of 1,000 patients who underwent surgery alone and 390 patients who received postoperative ACRT. The ACRT group had better results than the surgery-along group in terms of 3-year DFS rate (46% vs. 36%, respectively) and median DFS time (30.6 months vs. 17.6 months, respectively) (P=0.006). Moreover, the 2-year local recurrence-free rates were 87% and 77%, respectively (P=0.003). These results suggest that postoperative ACRT is more effective in improving postoperative recurrence compared to surgery alone for patients with locally advanced ESCC.

Both NCRT and postoperative ACRT have been shown to improve patient survival outcomes and reduce postoperative recurrence compared to surgery alone. However, there is currently limited research comparing the recurrence patterns associated with these two treatment strategies. The aim of this study was to compare the recurrence patterns and survival outcomes of patients with ESCC receiving NCRT and postoperative ACRT, as well as analyze the clinical factors that influence the prognosis of patients experiencing recurrence. The findings are intended to provide insights for the development of effective tertiary prevention strategies and subsequent treatments.

## 2 Methods

### 2.1 Patients

This study retrospectively collected clinical data from patients who underwent esophagectomy at Sichuan Cancer Hospital and Institute (Chengdu, China) between January 2018 and December 2020, based on the following selection criteria. The inclusion criteria were: 1) pathological confirmation of squamous cell carcinoma located in the thoracic segment by preoperative endoscopic biopsy; 2) no history of other malignancies; 3) preoperative clinical staging according to the 8th edition of the American Joint Committee on Cancer as cT2N1-2M0 or cT3-4aN0-2M0; 4) presence of tumors assessed by imaging as resectable and eligible for curative esophagectomy; 5) treatment with NCRT combined with surgery, or surgery combined with postoperative ACRT; 6) complete clinical medical records; and 7) a completed postoperative follow-up. The exclusion criteria were: 1) absence of squamous carcinoma as indicated by preoperative endoscopic biopsy; 2) history of recurrent cancer or other malignancies; 3) preoperative imaging examination suggesting unresectability or distant metastases; 4) perioperative mortality; 5) incomplete medical records; and 6) loss to follow-up.

### 2.2 Statistical analyses

Statistical analyses were performed using SPSS version 26.0 (IBM, Armonk, NY, USA). Categorical variables were compared using chi-squared and Fisher’s exact tests. The Kaplan–Meier method was employed to evaluate OS and DFS. The log-rank test was used to compare the survival rates between the two groups. Univariate and multivariate survival analyses were conducted using the Cox proportional hazards regression model to calculate hazard ratios and 95% confidence intervals. Initially, univariate regression analysis was performed to examine the relationships between all independent variables and the dependent variable. Prognostic factors with P-values <0.50 were subsequently included in the multivariate analysis.

All patients included in the final analysis underwent propensity score matching (PSM); using the postoperative ACRT group as the reference, a 1:1 nearest neighbor matching method was employed to match with the NCRT group. The matching process considered a total of 10 covariates (i.e., sex, age, number of surgical lymphadenectomy fields, surgical approach, tumor location, margin status, number of lymph nodes dissected, clinical T staging, clinical N staging, and clinical TNM staging), with a caliper value set at 0.05.

### 2.3 Treatment approaches

Surgical approaches were minimally invasive surgery and open surgery. The minimally invasive procedure involved thoracoscopic and laparoscopic radical resection of esophageal cancer through three paths (i.e., cervical, thoracic and abdominal) (McKeown), whereas the open surgical technique employed an upper abdomen-right thoracic radical esophagectomy (Ivor–Lewis). For patients who were diagnosed with positive cervical lymph nodes through preoperative neck ultrasound and biopsy, doctors usually choose to perform three-field lymph node dissection. Neoadjuvant chemotherapy consisted of platinum-based two-drug combination regimens and monotherapy with tegafur: paclitaxel plus platinum (n=70); docetaxel plus platinum (n=1); tegafur (n=1); and oxaliplatin plus fluorouracil (n=2). The median number of chemotherapy cycles was two (range: 1–4). Postoperative adjuvant chemotherapy included both platinum-based two-drug combination regimens and monotherapy with tegafur: paclitaxel plus platinum (n=55); tegafur (n=16); and docetaxel plus platinum (n=3), with a median of two cycles (range: 1–4). The median dose of preoperative radiotherapy was 40 (range: 20–56) Gy, delivered in fractions of 1.8–2.0 Gy each. The median dose of postoperative radiotherapy was 50 (range: 14.4–60) Gy, also delivered in fractions of 1.8–2.0 Gy each. Radical esophagectomy was performed in patients without evident disease progression or contraindications to surgery during a follow-up of 4–8 weeks after completing NCRT. ACRT was commenced 4–6 weeks postoperatively.

### 2.4 Observation indicators and follow-up

OS was defined as the interval from the date of first treatment to patient death or the last follow-up date. DFS was defined as the duration from the date of surgery to the earliest occurrence of relapse or death. Recurrences were categorized as local recurrences or distant metastases based on histological, cytological, or clear imaging evidence. Local recurrence was defined as recurrence within the esophagus, at the anastomosis site, or in regional lymph nodes, with the extent of regional lymph nodes classified according to the 8th edition of the American Joint Committee on Cancer. Distant metastasis was defined as recurrence in non-regional lymph nodes, hematogenous spread to solid organs, or recurrence in the pleural or peritoneal cavities; non-regional lymph nodes included cervical lymph nodes. Early and late recurrences were defined as recurrences occurring within and after 1 year post surgery, respectively. Follow-up was commenced 3 months postoperatively and was conducted every 6 months through various means, such as telephone calls, text messages, or in-person evaluations. The follow-up assessment included symptom evaluation, hematological tests, imaging studies, and endoscopic examinations to detect the presence of recurrence and/or metastasis postoperatively.

## 3 Results

### 3.1 Patient characteristics

A total of 267 patients with ESCC were included in this study (NCRT group: n=181; postoperative ACRT group: n=86). Prior to PSM, comparisons between the NCRT group and the postoperative ACRT group revealed that the former had a higher proportion of negative surgical margins (96.7% vs. 89.5%, respectively; P=0.018), fewer patients with >20 lymph nodes dissected (33.7% vs. 62.8%, respectively; P=0.001), a greater percentage of clinical N stage-positive cases (97.8% vs. 91.9%, respectively; P=0.023), and more patients classified as clinical TNM stage IV (22.7% vs. 10.5%, respectively; P=0.007). Statistically significant differences were observed between the two groups regarding the surgical margin status, number of lymph nodes dissected, clinical N staging, and clinical TNM staging. After PSM, each group comprised 74 patients. There were no statistically significant differences found in the rates of negative surgical margins (91.9% vs. 94.6%, respectively; P=0.512), number of patients with >20 lymph nodes dissected (54.1% vs. 58.1%, respectively; P=0.619), frequency of clinical N stage-positive cases (95.9% vs. 95.9%, respectively; P=1.000), or number of patients classified as clinical TNM stage IV (14.9% vs. 10.8%, respectively; P=0.561) (**Table 1**).

**Table 1:** Baseline characteristics of patients.

	Before PSM				χ2	P	After PSM				χ2	P
	NCRT group		ACRT group				NCRT group		ACRT group			
Total (n)	181		86				74		74			
Age (%)					2.269	0.132					1.020	0.312
≤65	132	(72.9)	70	(81.4)			56	(75.7)	61	(82.4)		
>65	49	(27.1)	16	(18.6)			18	(24.3)	13	(17.6)		
Gender (%)					1.435	0.231					0.060	0.806
Male	161	(89.0)	72	(83.7)			65	(87.8)	64	(86.5)		
Female	20	(11.0)	14	(16.3)			9	(12.2)	10	(13.5)		
Surgical method (%)					0.432	0.511					0.118	0.731
MIE	164	(90.6)	80	(93.0)			70	(94.6)	69	(93.2)		
Open surgery	17	(9.4)	6	(7.0)			4	(5.4)	5	(6.8)		
Location (%)					1.457	0.483					0.136	0.934
Upper	21	(11.6)	7	(8.1)			8	(10.8)	7	(9.5)		
Middle	108	(59.7)	49	(57.0)			39	(52.7)	41	(55.4)		
Lower	52	(28.71)	30	(34.9)			27	(36.5)	26	(35.1)		
Field of lymph node dissection (%)					1.100	0.294					0.000	1
Two field	161	(89.0)	80	(93.0)			69	(93.2)	69	(93.2)		
Three field	20	(11.0)	6	(7.0)			5	(6.8)	5	(6.8)		
R0 esophagectomy (%)					5.621	0.018					0.429	0.512
R0	175	(96.7)	77	(89.5)			68	(91.9)	70	(94.6)		
R1/2	6	(3.3)	9	(10.5)			6	(8.1)	4	(5.4)		
No. of resected nodes (%)					2.119	0.001					0.247	0.619
<20	120	(66.3)	32	(37.2)			34	(45.9)	31	(41.9)		
≥20	61	(33.7)	54	(62.8)			40	(54.1)	43	(58.1)		
cT (%)					5.886	0.053					1.210	0.546
T2	9	(5.0)	3	(3.5)			3	(4.1)	2	(2.7)		
T3	132	(72.9)	74	(86.0)			59	(79.7)	64	(86.5)		
T4	40	(22.1)	9	(10.5)			12	(16.2)	8	(10.8)		
cN (%)					5.189	0.023					0.000	1.000
N0	4	(2.2)	7	(8.1)			3	(4.1)	3	(4.1)		
N+	177	(97.8)	79	(91.9)			71	(95.9)	71	(95.9)		
cSt (%)					9.974	0.007					1.156	0.561
II	4	(2.2)	7	(8.1)			4	(5.4)	3	(4.1)		
III	136	(75.1)	70	(81.4)			59	(79.7)	63	(85.1)		
IV	41	(22.7)	9	(10.5)			11	(14.9)	8	(10.8)		

### 3.2 Location of recurrence

After a median follow-up period of 43.0 months, 44 (59.5%) and 25 (33.8%) patients experienced recurrence in the NCRT group and the postoperative ACRT group, respectively. The difference in the recurrence rates between the two groups was statistically significant (P=0.002).

Of the 44 patients in the NCRT group who experienced recurrence, 26 had local recurrence (59.1%; 26/44). Among the local recurrence cases, only three occurred at the anastomosis site (4.1%; 3/74), while the remaining 24 cases were regional lymph node recurrences (32.4%; 24/74). Regarding the distribution of regional lymph node recurrences, 19 patients had mediastinal lymph node recurrences (43.2%; 19/44), while nine patients had abdominal lymph node recurrences (20.5%; 9/44). In contrast, the postoperative ACRT group had a significantly lower rate of local recurrence compared to the NCRT group (16.2% vs. 35.1%, respectively; P=0.008). In the ACRT group, the rates of anastomosis and regional lymph node recurrence were 1.4% (1/74) and 16.2% (12/74), respectively. The rate of regional lymph node recurrence was lower in the ACRT group than the NCRT group (16.2% vs. 32.4%, respectively; P=0.022). Regarding the distribution of regional lymph node recurrences, five patients experienced mediastinal lymph node recurrences, accounting for 20.0% (5/25) of all patients who experienced recurrence with a recurrence rate of 6.8% (5/74). There was a statistically significant difference in the rate of mediastinal lymph node recurrence between two groups (6.8% vs. 25.7%, respectively; P=0.002). The rate of abdominal lymph node recurrence was slightly higher in the NCRT group (12.2%; 9/74) than the ACRT group (9.5%; 7/74), without statistical significance (P=0.597).

In the NCRT group, there were 31 cases of distant metastasis (70.5%; 31/44). Among the distant metastasis cases, only three were non-regional lymph node recurrences (6.8%, 3/44), while the remaining 28 were hematogenous metastases (63.6%; 28/44). The most common organ for hematogenous metastases was the lung, accounting for 27.3% (12/44) of all recurrences in this group, followed by the liver (25%; 11/44). In the postoperative ACRT group, there were 20 (80%; 20/25) cases of distant metastasis; there was no statistically significant difference in the distant metastasis rate between the two groups (27.0% vs. 41.9%, respectively; P=0.057). Regarding the types of distant metastasis, four cases (16.0%; 4/25) had non-regional lymph node metastases and 16 cases (64.0%; 16/25) had hematogenous metastases. In the ACRT group, the hematogenous metastases rates was lower compared to the NCRT group (21.6% vs. 47.8%, respectively; P=0.031). In the ACRT group, the organ with the highest rate of hematogenous metastasis was the lung (28.0%; 7/25), followed by the bones (20.0%; 5/25) and liver (20.0%; 5/25) (**Table 2**).

**Table 2:** Type and location of recurrence in NCRT group and ACRT group.

	NCRT group n=44 (59.5%)	ACRT group n=25 (33.8%)	P 0.002
Local recurrences	26 (35.1%)	12 (16.2%)	0.008
Anastomosis	3 (4.1%)	1 (1.4%)	0.612
Regional lymph node	24 (32.4%)	12 (16.2%)	0.022
Mediastinal lymph node	19 (25.7%)	5 (6.8%)	0.002
Abdominal lymph node	9 (12.2%)	7 (9.5%)	0.597
Distant metastasis	31 (41.9%)	20 (27.0%)	0.057
Non-regional lymph node	3 (4.1%)	4 (5.4%)	1.000
Hematogenous metastases	28 (37.8%)	16 (21.6%)	0.031
Lung	12 (16.2%)	7 (9.5%)	0.219
Bone	6 (8.1%)	5 (6.8%)	0.754
Liver	11 (14.9%)	5 (6.8%)	0.112
Kidney	2 (2.7%)	2 (2.7%)	1.000
Brain	1 (1.4%)	1 (1.4%)	1.000
Pleura	2 (2.7%)	1 (1.4%)	1.000
Combined recurrence	14 (18.9%)	6 (8.1%)	0.054

### 3.3 Time and frequency of recurrence

The majority (82.6%; 57/69) of recurrences occurred within the first 2 years following surgery. In the NCRT group, the majority of local recurrences occurred in year 1 and year 2 (38.5%; 10/26). In the postoperative ACRT group, the highest local recurrence rate was observed in year 1, with six patients experiencing recurrence (50.0%). The difference in the rates of early (year 1) local recurrence between the two groups was not statistically significant (P=0.725). Regarding distant metastasis, the highest rates were recorded in the first year following surgery in both the NCRT and postoperative ACRT groups (16 cases; 51.6% vs. nine cases; 45.0%). The difference in the rate of early metastasis between the two groups was not statistically significant (P=0.771). Overall, for all types of recurrence, there was no statistically significant difference in the rate recorded in year 1 between the two groups (45.5% vs. 48.0%, respectively; P=0.839) (**Table 3**).

**Table 3:** Time and frequency of recurrence in NCRT group and ACRT group.

	n	≤12.0 month	12.1–24.0 month	24.1–36.0 month	≥36.1 month
Local recurrences					
NCRT group	26	10 (38.5 %)	10 (38.5%)	4 (15.4%)	2 (7.7%)
ACRT group	12	6 (50.0%)	3 (25.0%)	1 (8.3%)	2 (16.7%)
Distant metastasis					
NCRT group	31	16 (51.6%)	12 (38.7%)	2 (6.5%)	1 (3.2%)
ACRT group	20	9 (45.0%)	7 (35.0%)	0 (0%)	4 (20.0%)
Total					
NCRT group	44	20 (45.5%)	16 (36.4%)	5 (11.4%)	3 (6.8%)
ACRT group	25	12 (48.0%)	9 (36.0%)	1 (4.0%)	3 (12.0%)

### 3.4 Survival status

**Figure 1:**
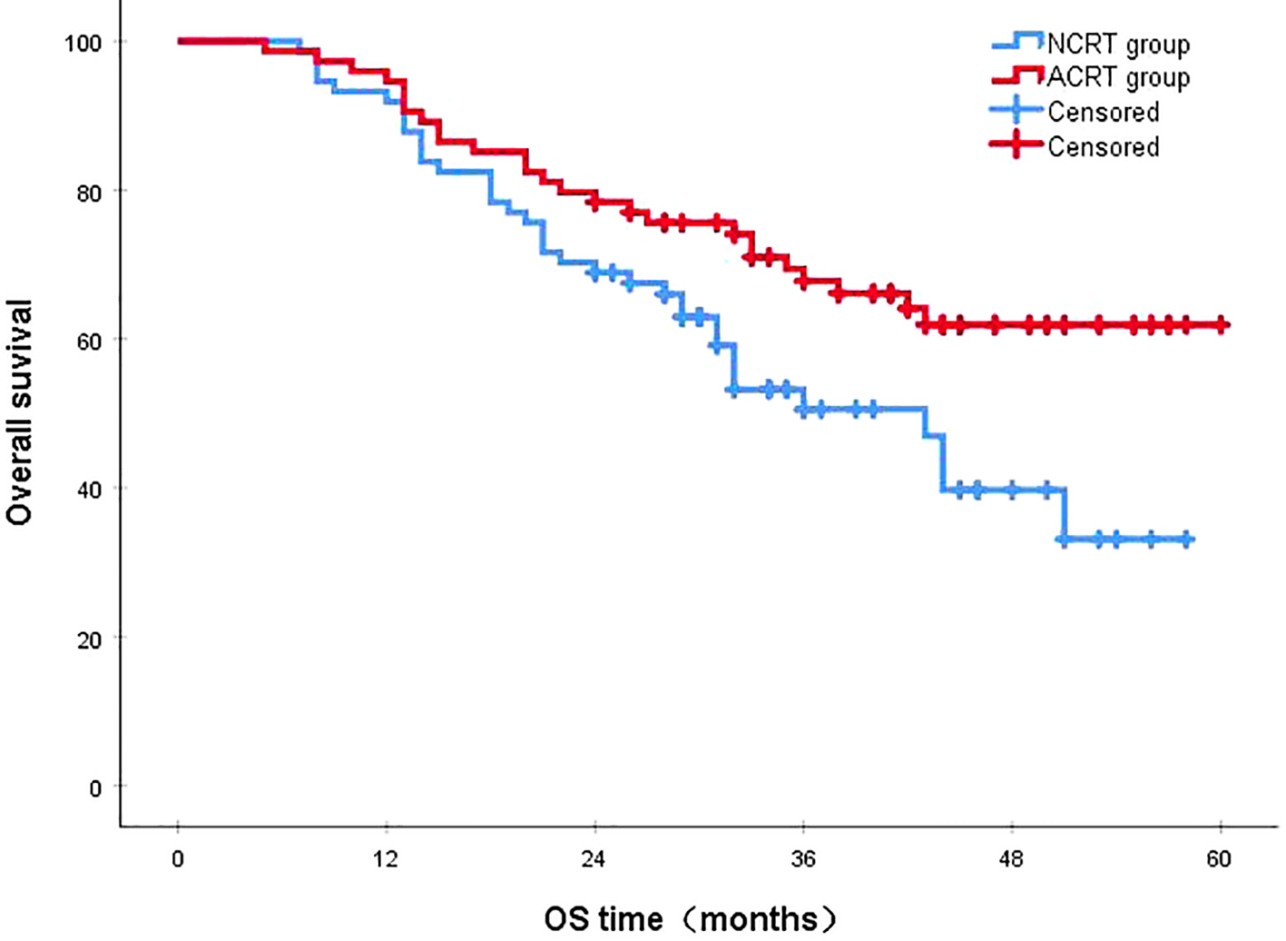
The 3-year survival rates for the NCRT and postoperative ACRT groups were 50.6% and 67.8%, respectively (P=0.019). ACRT, adjuvant chemoradiotherapy; NCRT, neoadjuvant chemoradiotherapy; OS, overall survival.

**Figure 2:**
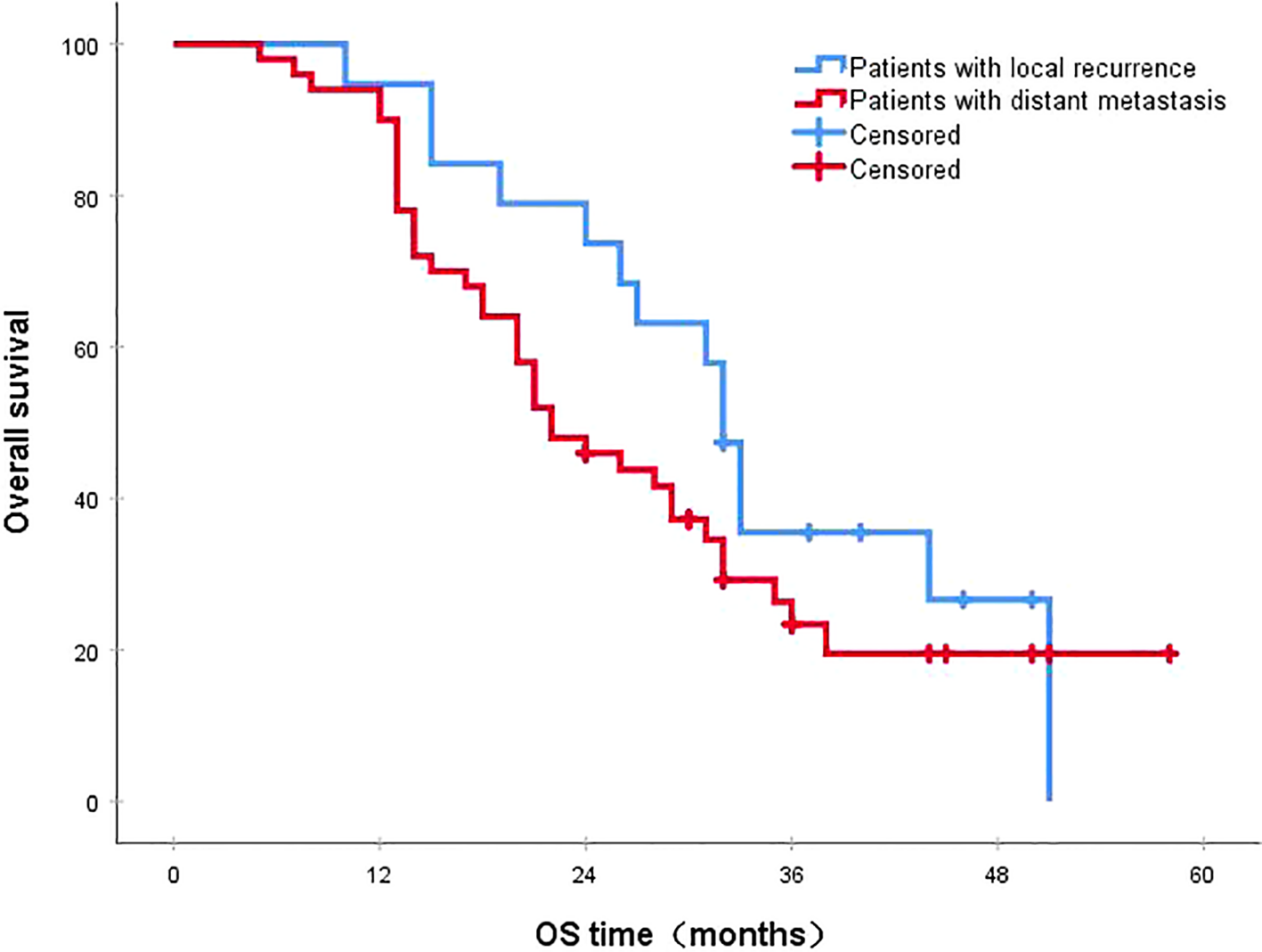
The 3-year survival rates for patients with local recurrence and distant metastasis were 35.5% and 23.4%, respectively (P=0.216). OS, overall survival.

**Figure 3:**
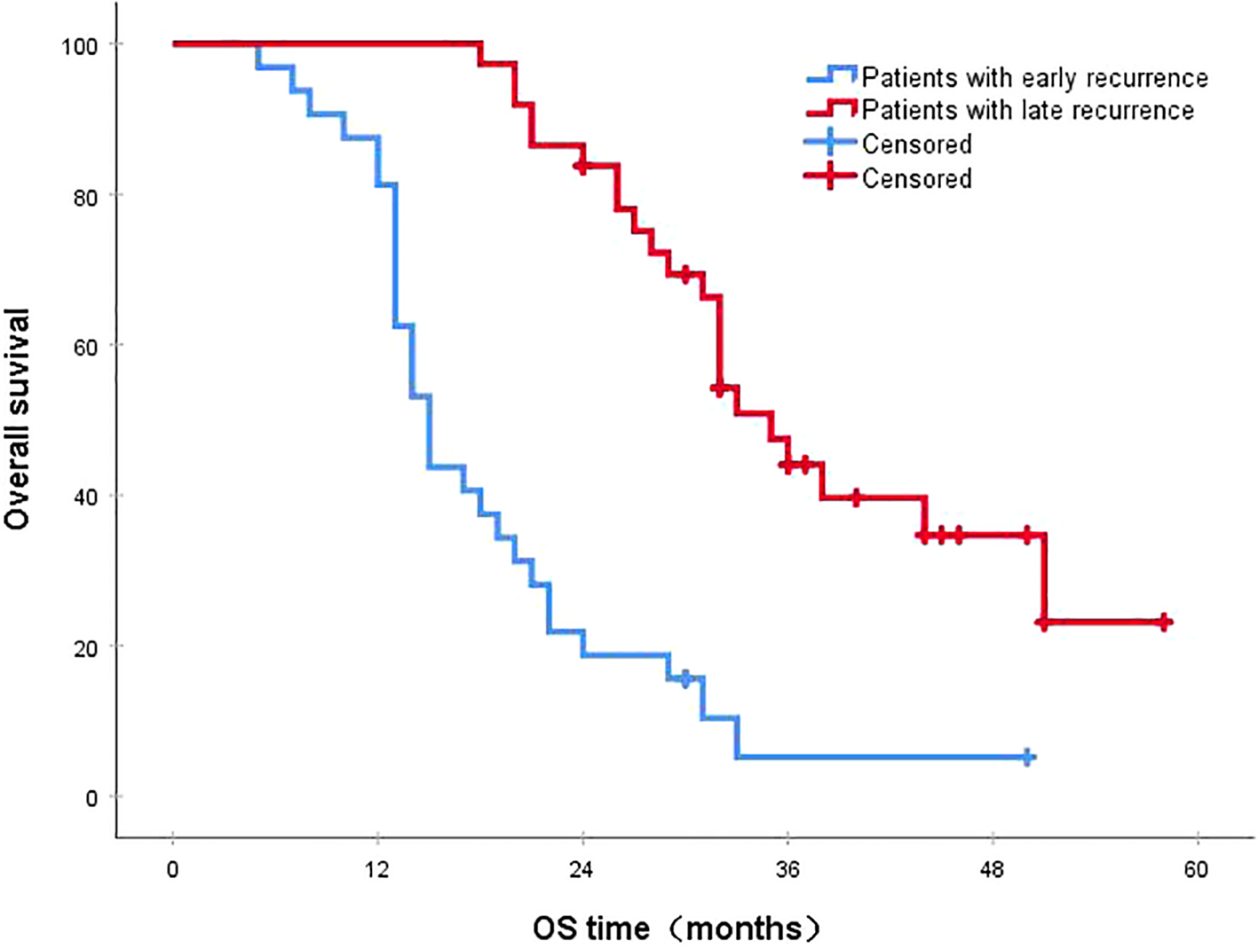
The 3-year survival rates for patients with early and late recurrences were 5.2% and 44.1%, respectively (P<0.001). OS, overall survival.

**Figure 4:**
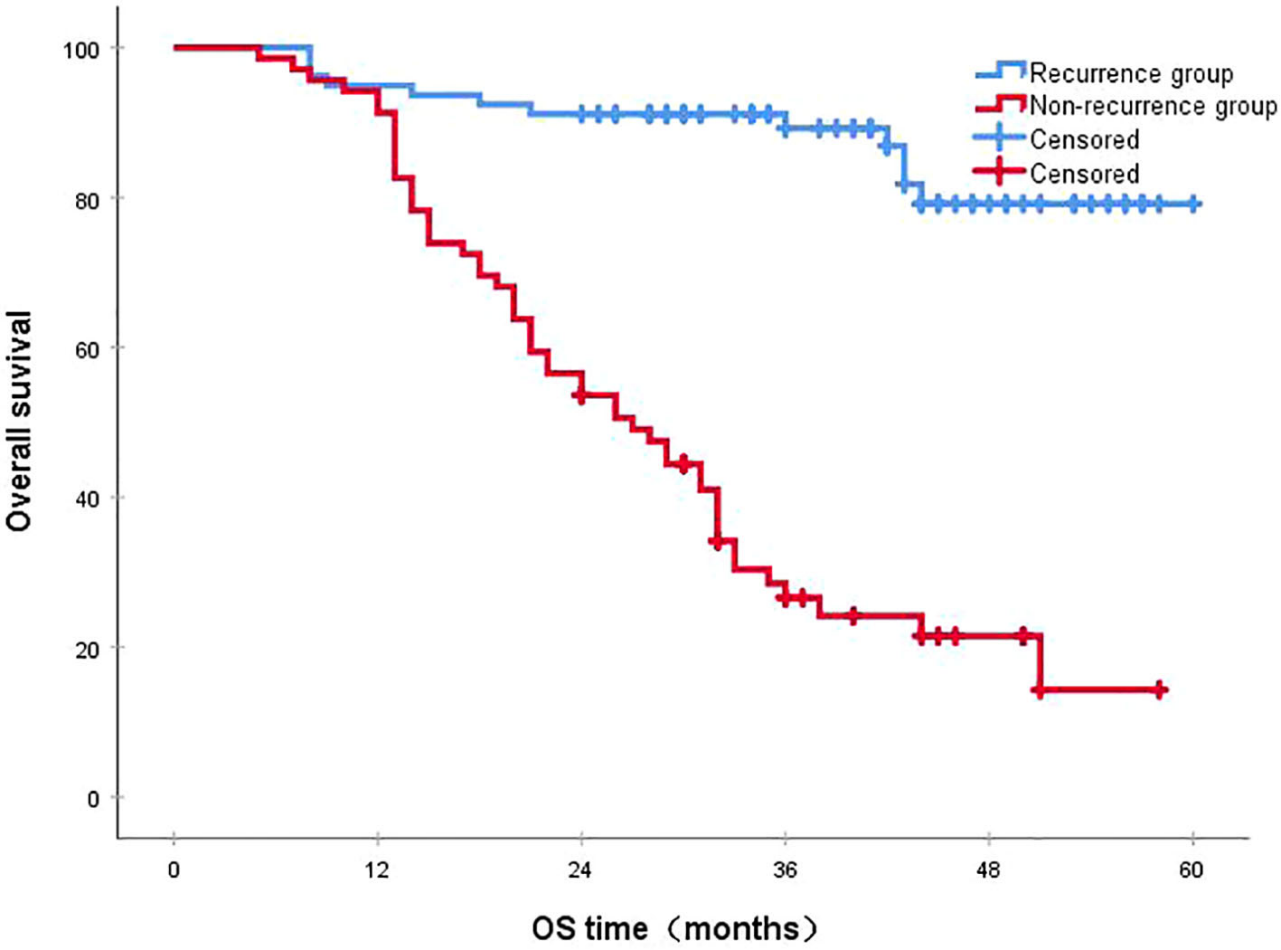
The 3-year survival rates in the recurrence and non-recurrence groups were 26.6% and 89.2%, respectively (P<0.001). OS, overall survival.

**Figure 5:**
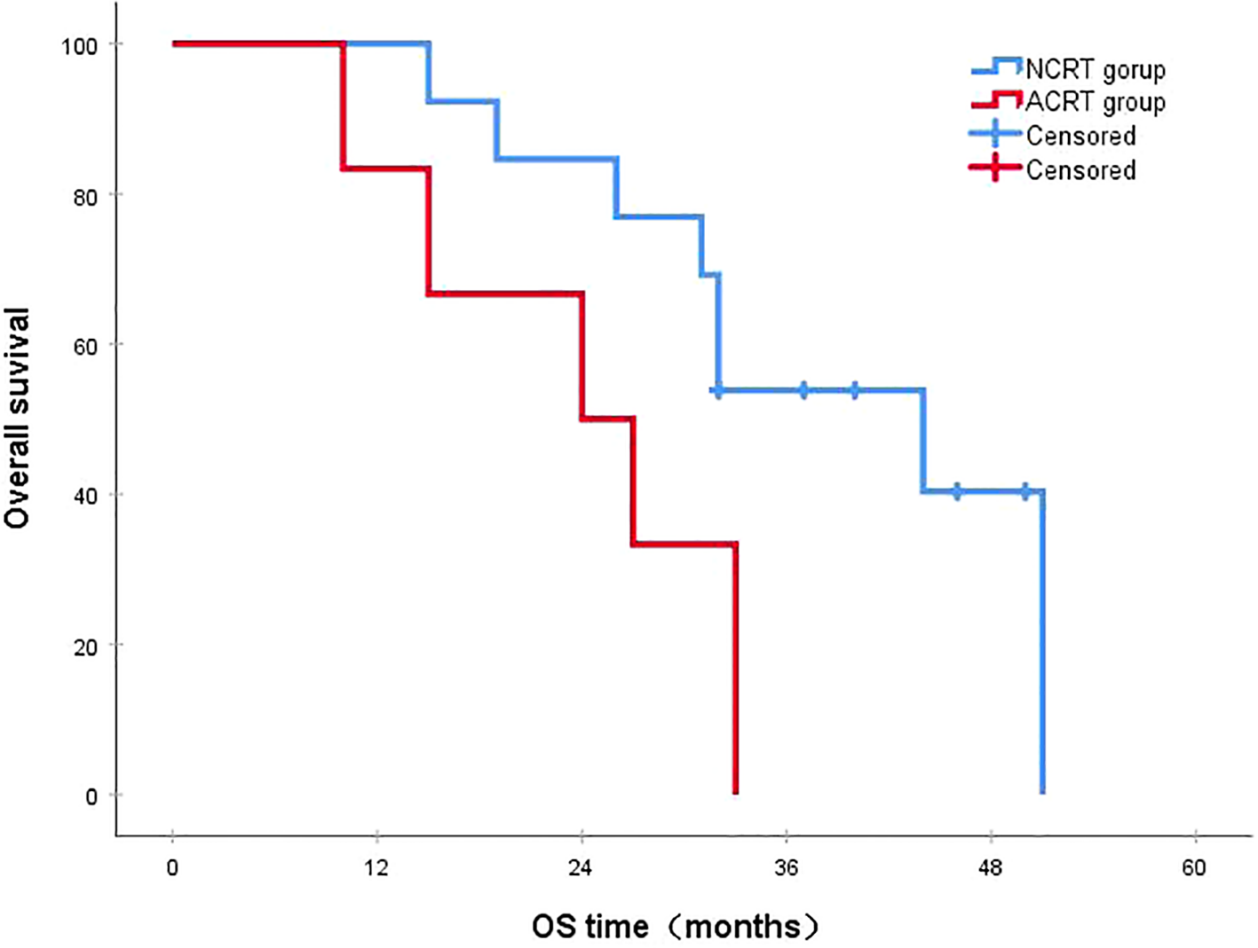
In patients with local recurrence, the 3-year survival rates in the NCRT and ACRT groups were 53.8% and 0%, respectively (P=0.029). ACRT, adjuvant chemoradiotherapy; NCRT, neoadjuvant chemoradiotherapy; OS, overall survival.

**Figure 6:**
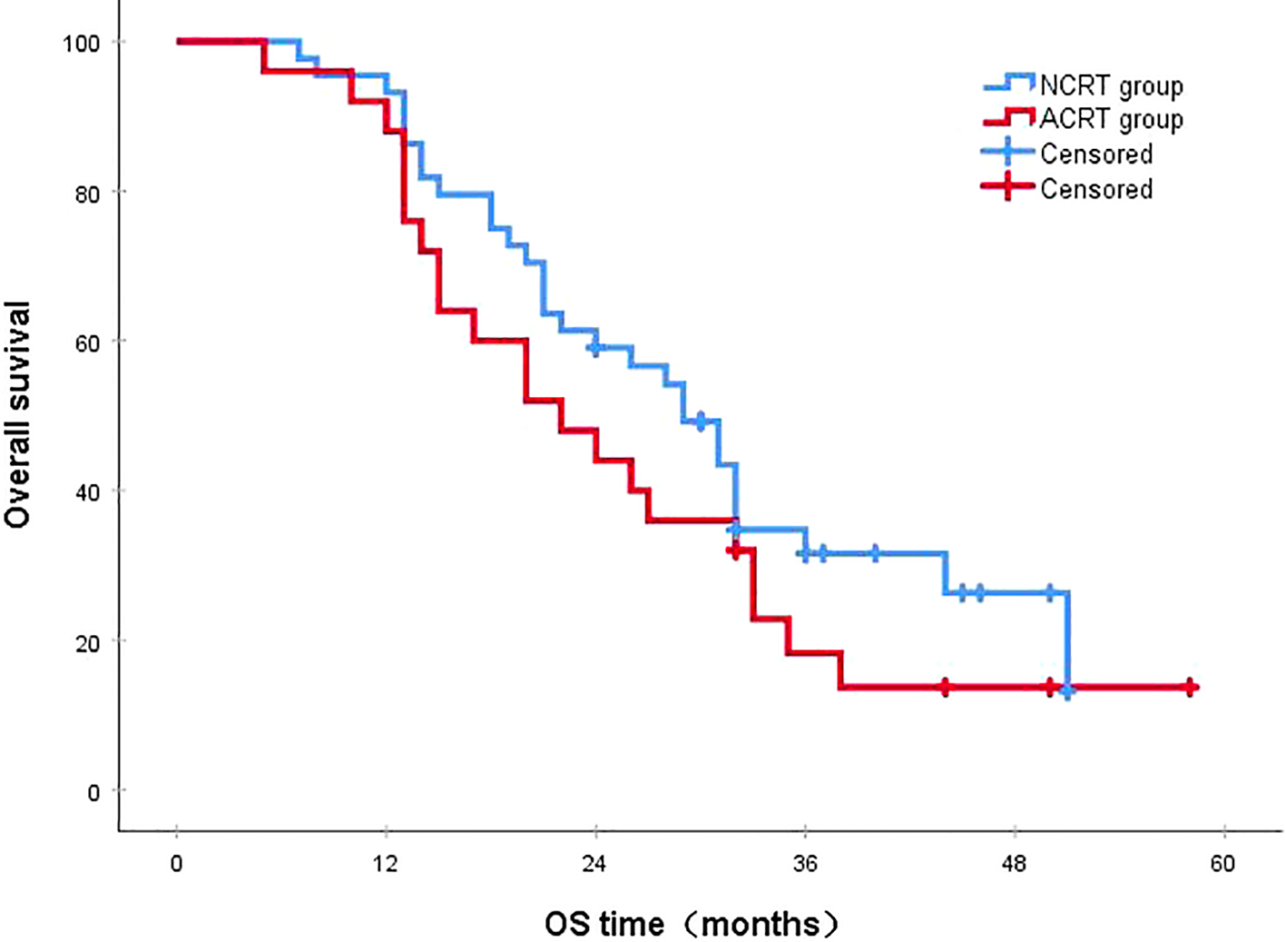
In the overall recurrence population, the 3-year survival rates in the NCRT and ACRT groups were 18.3% and 31.6%, respectively (P=0.216). ACRT, adjuvant chemoradiotherapy; NCRT, neoadjuvant chemoradiotherapy; OS, overall survival.

**Figure 7:**
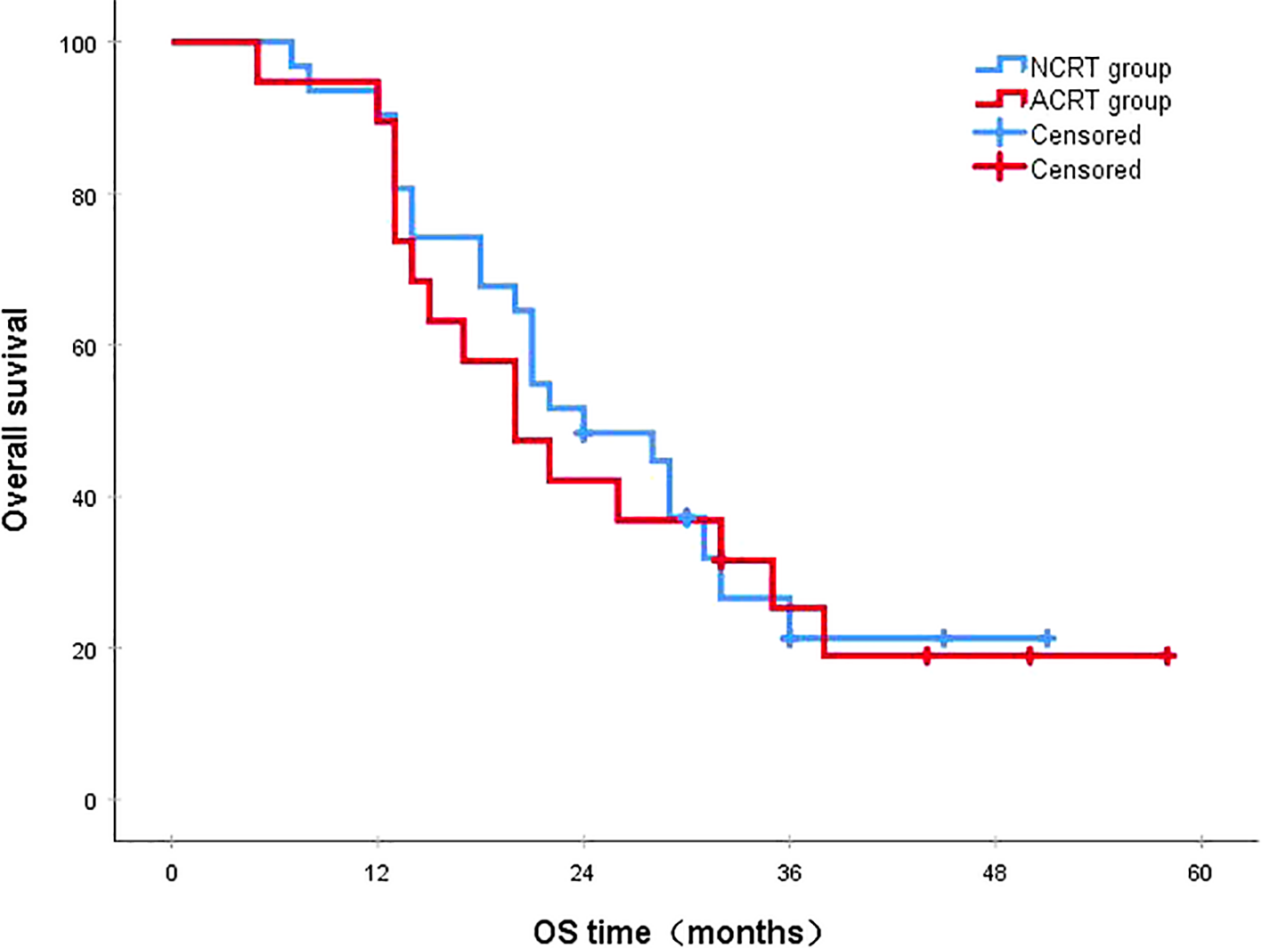
In patients with distant metastasis, the 3-year survival rates in the NCRT and ACRT groups were 21.3% and 25.3%, respectively (P=0.787). ACRT, adjuvant chemoradiotherapy; NCRT, neoadjuvant chemoradiotherapy; OS, overall survival.

**Figure 8:**
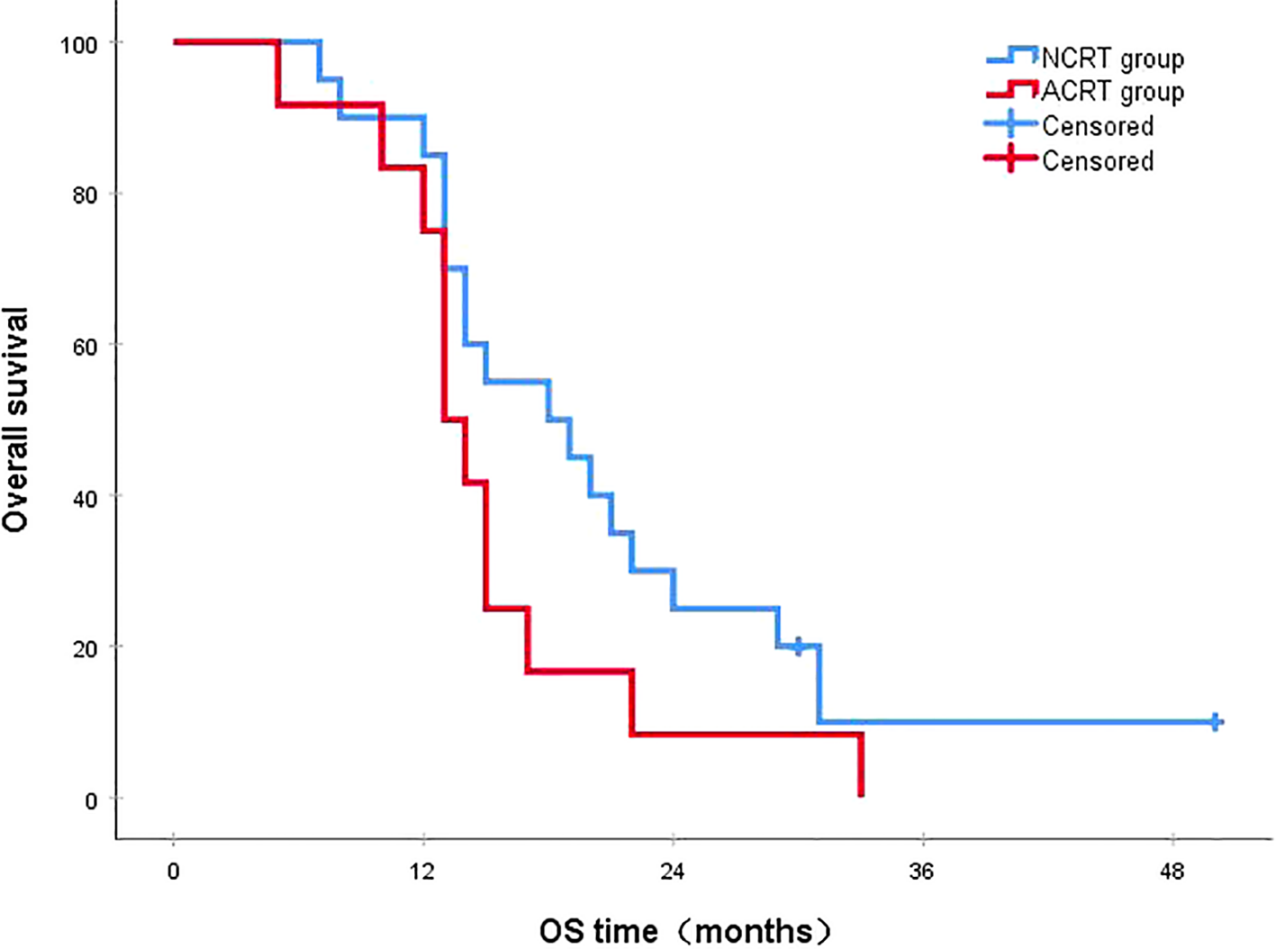
In patients with early recurrence, the 3-year survival rates in the NCRT and ACRT groups were 10.0% and 0%, respectively (P=0.136). ACRT, adjuvant chemoradiotherapy; NCRT, neoadjuvant chemoradiotherapy; OS, overall survival.

**Figure 9:**
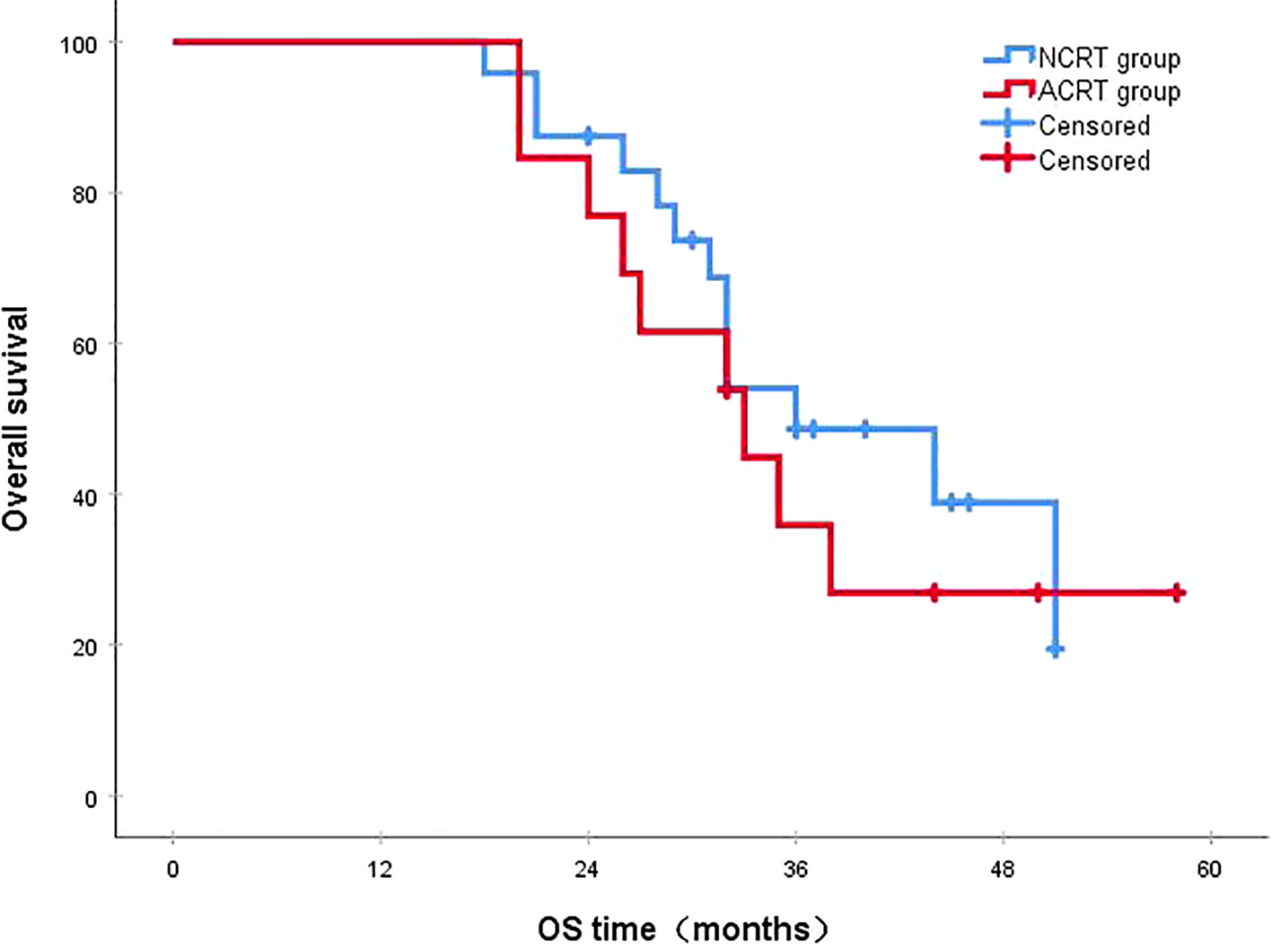
In patients with late recurrence, the 3-year survival rates in the NCRT and ACRT groups were 48.6% and 35.9%, respectively (P=0.528). ACRT, adjuvant chemoradiotherapy; NCRT, neoadjuvant chemoradiotherapy; OS, overall survival.

**Figure 10:**
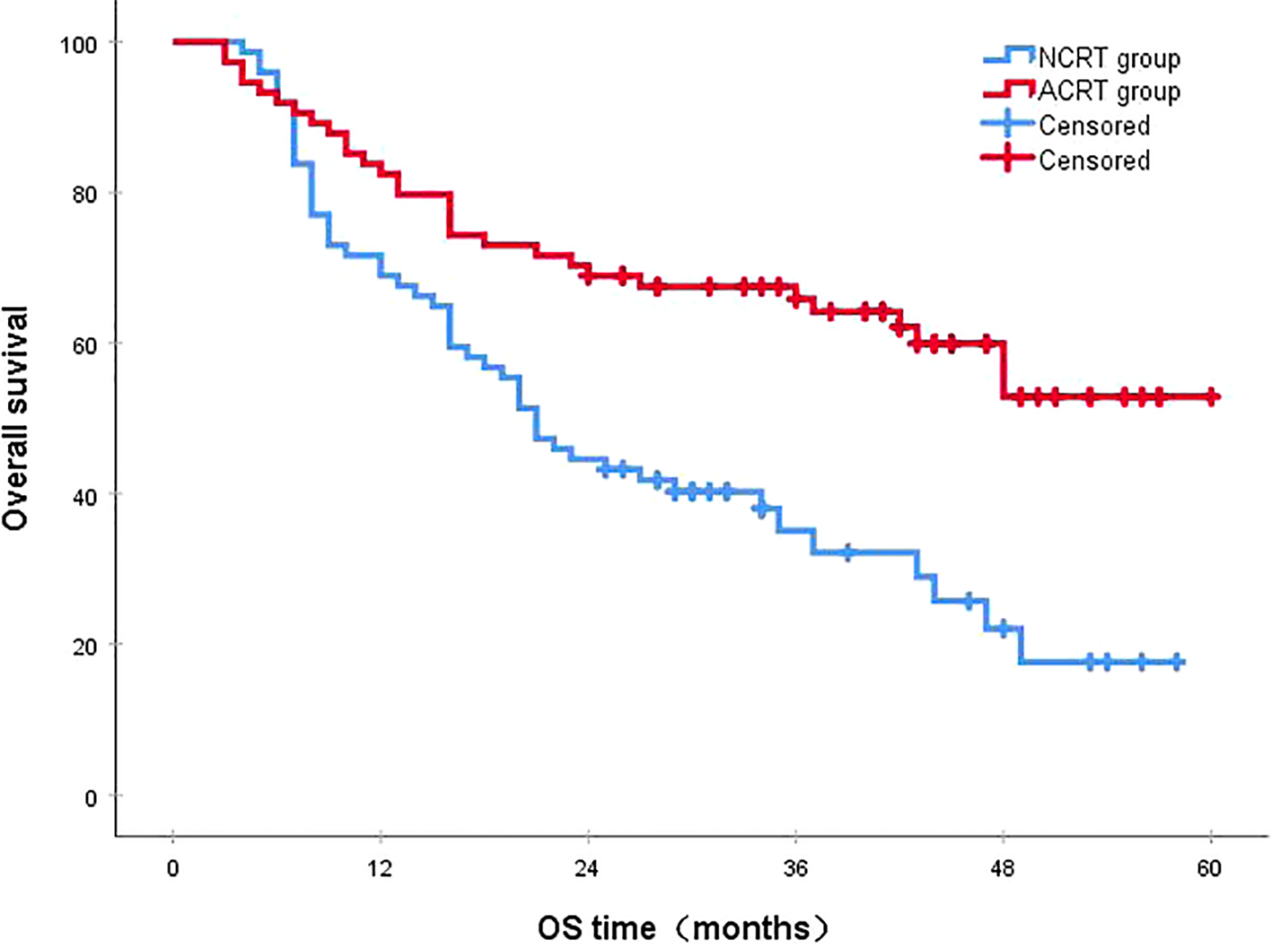
Disease-free survival (DFS). The 3-year DFS rates in the NCRT and postoperative ACRT groups were 51.4% and 65.8%, respectively (P<0.001). ACRT, adjuvant chemoradiotherapy; NCRT, neoadjuvant chemoradiotherapy; OS, overall survival.

Following a median follow-up period of 43 months, the 3-year survival rates for the NCRT group and the postoperative ACRT group were 50.6% and 67.8%, respectively. The difference between the two groups was statistically significant (P=0.019) (**Figure 1**). Among the overall recurrence population, the median survival time for patients with local recurrence was 32.0 months, with a 3-year survival rate of 35.5%. The median survival time for patients with distant metastasis was 22.0 months, and the 3-year survival rate was 23.4%. The difference between the two groups was not statistically significant (P=0.216) (**Figure 2**). Patients with early and late recurrences had median survival times of 15.0 months and 35.0 months, respectively, and 3-year survival rates of 5.2% and 44.1%, respectively; the differences between the two groups were statistically significant (P<0.001) (**Figure 3**). In the overall recurrence population, the median survival time for the recurrence group was 27 months, while that for the non-recurrence group was not reached. The 3-year survival rate for the non-recurrence group was 89.2% compared to 26.6% for the recurrence group, demonstrating a statistically significant difference (P<0.001) (**Figure 4**). Further subgroup analysis demonstrated that, in patients with local recurrence, the NCRT group had better survival outcomes compared to the ACRT group (53.8% vs. 0%, respectively; P=0.029) (**Figure 5**). However, this difference was not observed in the overall recurrence population (18.3% vs. 31.6%, respectively; P=0.216) (**Figure 6**), patients with distant metastasis (21.3% vs. 25.3%, respectively; P=0.787) (**Figure 7**), patients with early recurrence (10.0% vs. 0%, respectively; P=0.136) (**Figure 8**), or patients with late recurrence (48.6% vs. 35.9%, respectively; P=0.528) (**Figure 9**). The ACRT group had a better 3-year DFS rate compared to the NCRT group (65.8% vs. 51.4%, respectively; P<0.001) (**Figure 10**).

### 3.5 Univariate and multivariate analyses of prognosis

After PSM, in the NCRT group, several factors (i.e., sex, age, surgical method, margin status, number of lymph nodes dissected, pathological T stage, and pathological N stage) were included in the univariate analysis. The results indicated that only pathological N stage was a prognostic risk factor affecting survival. Variables from the univariate analysis with P<0.50 (i.e., sex, pathological T stage, and pathological N stage) were entered into a multivariate Cox regression model. The results showed that pathological N stage was a risk factor influencing OS prognosis in the NCRT group (**Table 4**).

**Table 4:** Univariate and multivariate Cox analysis of the overall survival prognosis of patients in NCRT group.

	n	Univariate analysis		P	Multivariate analysis	P
		HR (95% CI)			HR (95% CI)	
Gender				0.264		0.166
Male	65	1.000			1.000	
Female	9	0.440	(0.105~1.855)		0.339(0.073~1.568)	
Age				0.578		
>65	18	1.000				
≤65	56	0.761	(0.290~1.995)			
Surgical method				0.710		
MIE	70	1.000				
Open surgery	4	1.460	(0.198~10.773)			
R0 esophagectomy				0.541		
R0	68	1.000				
R1/2	6	1.570	(0.370~6.667)			
No. of resected nodes				0.638		
≥20	40	1.000				
<20	34	0.832	(0.387~1.790)			
pT						
T0	26	1.000			1.000	
T1	13	0.937	(0.294~2.988)	0.913	0.904 (0.280~2.918)	0.866
T2	18	1.230	(0.411~3.684)	0.711	1.133 (0.373~3.437)	0.826
T3	17	2.389	(0.919~6.210)	0.074	1.074 (0.356~3.234)	0.899
pN						
N0	50	1.000			1.000	
N1	19	3.377	(1.501~7.598)	0.003	3.185(1.363~7.441)	0.007
N2	5	5.915	(2.031~17.225)	0.001	7.51(1.962~28.771)	0.003

In the univariate analysis of OS prognosis for the postoperative ACRT group, pathological T staging and pathological N staging were identified as significant prognostic risk factors affecting survival. Univariate variables with P<0.50 (i.e., sex, age, surgical method, margin status, pathological T staging, and pathological N staging) were included in the multivariate analysis. The results indicated that margin status and pathological T staging were significant prognostic risk factors impacting survival (**Table 5**).

**Table 5:** Univariate and multivariate Cox analysis of the overall survival prognosis of patients in ACRT group.

	n	Univariate analysis		P	Multivariate analysis	P
		HR (95% CI)			HR (95% CI)	
Gender				0.102		0.163
Male	64	1.000			1.000	
Female	10	0.301	(0.071~1.271)		0.339 (0.074~1.548)	
Age				0.231		0.499
>65	13	1.000			1.000	
≤65	61	1.638	(0.731~3.670)		1.359 (0.558~3.310)	
Surgical method				0.491		0.825
MIE	69	1.000			1.000	
Open surgery	5	0.496	(0.068~3.649)		0.794 (0.102~6.166)	
R0 esophagectomy				0.179		0.029
R0	70	1.000			1.000	
R1/2	4	2.689	(0.636~11.369)		5.808 (1.194~28.265	
No. of resected nodes				0.866		
≥20	43	1.000				
<20	31	0.936	(0.431~2.033)			
pT						
T1	10	1.000			1.000	
T2	7	2.525	(0.229~27.857)	0.450	2.202 (0.180~26.966)	0.866
T3	55	5.537	(0.749~40.923)	0.094	4.937 (0.615~39.650)	0.826
T4	2	20.584	(2.116~200.243)	0.009	14.655 (1.263~170.041)	0.032
pN						
N0	10	1.000			1.000	
N1	40	1.947	(0.445~8.532)	0.377	2.334 (0.507~10.744)	0.276
N2	19	3.571	(0.781~16.327)	0.101	3.260 (0.679~15.644)	0.140
N3	5	5.698	(1.037~31.303)	0.045	3.895 (0.670~22.647)	0.130

## 4 Discussion

For locally advanced ESCC, which is associated with poor prognosis and high recurrence rates, a combined treatment approach involving radiotherapy, chemotherapy, and surgery is currently adopted in clinical practice. At present, NCRT is recommended as a standard treatment for locally advanced ESCC. In China, significant variations in treatment methods for esophageal cancer exist due to differences in economic levels and access to medical resources across regions. Data show that only 22.0% of patients received NCRT, while postoperative adjuvant treatment was administered to 43.5% of patients (21). Therefore, studies comparing NCRT and ACRT are warranted. This study represents the first known comparative analysis of these two treatment modalities. Our findings indicate that both NCRT and ACRT significantly improved the 3-year survival rate of patients (50.6% vs. 67.8%, respectively). Notably, among patients with local recurrence, NCRT demonstrated a significant improvement in the 3-year survival rate compared to ACRT (53.8% vs. 0%, respectively; P=0.029). The evidence from this study holds important implications for clinical practice and the design of future prospective clinical trials. In this study, the average radiation dose received by patients in the ACRT group was higher than that in the NCRT group (50 Gy vs 40 Gy). This may be because after receiving neoadjuvant treatment, the primary tumors and positive lymph nodes of the patients in the NCRT group were improved, resulting in a lower radiation dose received by this group compared to the postoperative treatment group.

The advantages of the NCRT treatment model include the elimination of systemic micro-metastases and reduction of tumor staging. These effects improve the R0 resection rate after surgery, facilitate radical resection, and ultimately enhance patient survival (22–24). Conversely, the advantage of postoperative adjuvant therapy lies in its more precise pathological staging compared to clinical staging before surgery. This allows for the selection of appropriate adjuvant treatment regimens based on staging to improve prognosis and minimize overtreatment of patients with early-stage disease (25–32).

The optimal sequence of combining surgery with chemoradiotherapy remains unclear, primarily due to a lack of high-quality controlled studies evaluating the efficacy and recurrence patterns of both treatment modalities. A retrospective study (33) included data from 1,647 patients with clinical stage II/III ESCC and used PSM to select 286 well-balanced pairs for comparison. The results demonstrated that the 3-year DFS rates in the NCRT group and the postoperative ACRT group were 38.7% and 30.2%, respectively (P=0.067). Among patients who achieved R0 resection, the 1-year recurrence-free rates were 74.8% and 67.6%, respectively (P=0.269). Furthermore, another retrospective study (34) directly compared NCRT with postoperative ACRT. The findings did not reveal statistically significant differences between the two groups regarding sex, age, disease stage, tumor grade and location, histopathology, and recurrence status. In this study, the rates of local recurrence rate and distant metastasis were higher in the NCRT group than the postoperative ACRT group; however, only the difference in local recurrence rate reached statistical significance. This may be attributed to a selection bias at this thoracic surgery center, where patients receiving postoperative ACRT were preferentially those with pathologically positive lymph nodes after esophagectomy. This preference may have improved the local recurrence rate. The results of this study indicate that recurrences predominantly occurred within the first 2 years (82.6%), suggesting a need to increase the frequency of follow-up examinations during the first 2 years post surgery. Among the types of local recurrence, mediastinal lymph node recurrence accounted for 43.2% of recurrences in the NCRT group; the recurrence rate was higher in the NCRT group than the postoperative ACRT group (25.7% vs. 6.8%, respectively; P=0.002). This finding highlights the need for increased attention to mediastinal lymph node recurrence during follow-up examinations for patients receiving neoadjuvant therapy. In addition, it suggests that improving NCRT strategies could mitigate the rate of such recurrences, thereby enhancing postoperative outcomes for these patients. Regarding the types of distant metastasis, the lungs (27.3%) and liver (25.0%) were the top two organs in which hematogenous metastasis occurred in the NCRT group, consistent with the findings of the NEOCRTEC5010 study. In contrast, the organs in which hematogenous metastasis occurred more frequently in the postoperative ACRT group were the lungs (28.0%), bones (20.0%), and liver (20.0%), aligning with the results obtained from the surgery-only group in the NEOCRTEC5010 study. This further indicates potential inadequacies in ACRT regarding the control of distant metastases.

Survival analysis in this study indicated that ACRT led to better survival outcomes than NCRT (3-year OS rates of 67.8% vs. 50.6%, respectively: P=0.019; DFS: P<0.001) in patients with esophageal cancer, contrary to the findings reported by Lv et al. (35). This discrepancy may be attributed to the fact that, in this study, the proportions of patients with positive lymph nodes and those with more advanced TNM staging were higher in the NCRT group. Additionally, patients in the ACRT group received a higher intensity of radiotherapy.

In the present study, the median survival times for the local recurrence group and distant metastasis group were 32.0 months and 22.0 months, respectively. Moreover, the 3-year survival rates were 35.5% and 23.4%, respectively, without statistically significant difference (P=0.216). This suggests that local recurrence and distant metastasis have comparable impacts on survival outcomes. Compared to the late recurrence group, the early recurrence group exhibited significantly shorter median survival (35.0 months vs. 15.0 months, respectively) and lower 3-year survival rates (44.1% vs. 5.2%, respectively) (P<0.001), indicating a poorer prognosis for patients experiencing early recurrence. Additionally, the survival prognosis was significantly lower for the recurrence group than the non-recurrence group (P<0.001), corroborating the findings of the NEOCRTEC5010 study. Furthermore, subgroup analysis showed that NCRT resulted in better survival outcomes compared to ACRT only in patients with local recurrence (53.8% vs. 0%, respectively; P=0.029). However, such a difference was not observed in the overall recurrence population, patients with distant metastasis, patients with early recurrence, or patients with late recurrence. This may suggest no significant difference between NCRT and ACRT in improving the prognosis of patients with recurrence. However, it is of note that due to the small sample size of this subgroup, the extrapolation of this result is limited. More large-sample prospective clinical studies are needed in the future to further prove this point.

The results of the multifactorial analysis in this study indicated that pathological N staging was an independent risk factor affecting OS prognosis in patients from the NCRT group, consistent with the findings of the NEOCRTEC5010 study. For the postoperative ACRT group, margin status and pathological T staging were identified as independent risk factors influencing OS, indicating that later pathological T staging and non-R0 resections adversely affect patient survival outcomes. Additionally, in the multifactorial analysis of patients with postoperative pathological lymph node positivity, sex and treatment regimen were identified as independent risk factors affecting recurrence-free survival. These observations suggest that patients in the NCRT group with postoperative positive lymph nodes had a poorer prognosis compared to their counterparts in the postoperative ACRT group.

This study had several limitations. Firstly, Due to the preference of surgeons, there exists a selection bias at this thoracic surgery center regarding patients with newly diagnosed esophageal cancer. Data prior to PSM showed that after completing clinical staging evaluations for new patients, those with advanced N staging or more advanced TNM staging (particularly stage IV) were more likely to receive NCRT. Even though we used PSM, we still can’t completely avoid these potential biases. Secondly, this study was retrospective and the sample size was relatively small. Finally, there was inconsistency in the chemotherapy regimens and radiation doses used preoperatively and postoperatively. The neoadjuvant chemotherapy regimens included combinations such as paclitaxel + platinum and oxaliplatin + fluorouracil, while the adjuvant chemotherapy regimens included paclitaxel + platinum, docetaxel + platinum, and tegafur, which may introduce bias into the results.

In conclusion, the results of this study indicate that, compared to NCRT, postoperative ACRT can also bring survival benefits to patients with ESCC. Recurrence primarily occurred within the first 2 years following esophagectomy. The recurrence rate of mediastinal lymph nodes was higher in the NCRT group compared to the postoperative ACRT group. Patients with early recurrence had worse survival outcomes than those with late recurrence. There were no significant differences found in the impact of local recurrence and distant metastasis on survival. Pathological N staging was identified as an independent risk factor affecting OS prognosis in the NCRT group. Margin status and pathological T staging were independent risk factors influencing OS in the postoperative ACRT group. The differences in recurrence patterns, sites, and frequencies between the treatment groups could provide insights into risk-based strategies for subsequent treatment and monitoring. With the ongoing accumulation of data and the extension of survival follow-up periods, alongside advancements in minimally invasive and robot-assisted surgical techniques, there is a growing need for well-designed randomized controlled trials to further validate these approaches.

## Data Availability

The raw data supporting the conclusions of this article will be made available by the authors, without undue reservation.
